# Serum NGAL and FGF23 may have certain value in early diagnosis of CIN

**DOI:** 10.1080/0886022X.2018.1487860

**Published:** 2018-10-03

**Authors:** Huihui Li, Zaixin Yu, Lu Gan, Ling Peng, Qiaoling Zhou

**Affiliations:** a Department of Nephropathy, Xiangya Hospital Central-South University, Changsha, Hunan410008, China;; b Department of Cardiology, Xiangya Hospital Central-South University, Changsha, Hunan410008, China

**Keywords:** Neutrophil gelatinase-associated lipocalin, fibroblast growth factor 23, contrast-induced nephropathy, percutaneous coronary intervention

## Abstract

**Aims**: This study aimed to assess whether neutrophil gelatinase-associated lipocalin (NGAL) and fibroblast growth factor 23 (FGF23) could be reliable biomarkers for early diagnosis of contrast-induced nephropathy (CIN).

**Methods**: 202 patients who underwent percutaneous coronary intervention (PCI) were included in the research. All subjects were divided into CIN group and non-CIN group. Serum NGAL and FGF23 were evaluated before and 0, 1, and 2 days after PCI. Serum levels of these two markers were compared intra-group and among groups. Receiver-operating characteristic (ROC) analysis and logistic regression models were conducted to assess the diagnostic performance of NGAL and FGF23 in detecting CIN.

**Results**: When compared with baseline values, serum levels of both NGAL and FGF23 in all subjects increased after PCI, and the values peaked 1 day after PCI, but the changing was greater in CIN group. There were obvious differences between two groups in serum NGAL after 1, 2 days, and similar differences present in serum FGF23 after 1 day. ROC analysis showed that the area under the curve (AUC) of relative values (percent change from the baseline) in NGAL after 1 day was 0.899 (95% CI: 0.834–0.964, *p* = .000), the optimum cutoff was 49% (sensitivity = 80%, specificity = 92.4%). And the AUC in FGF23 was 0.814 (95% CI: 0.733–0.894, *p* = .000), the optimum cutoff was 20% (sensitivity = 73.3%, specificity = 87.6%). Both serum NGAL and serum FGF23 could improve the clinical models in identifying CIN.

**Conclusions**: NGAL and FGF23 may have certain value in early diagnosis of CIN.

## Introduction

Acute kidney injury (AKI) is a dangerous and frequently occurring disease and its morbidity is gradually increasing. A systematic review [1] containing 154 studies showed that the pooled incidence rates of AKI were 21.6% in adults (95% CI, 19.3–24.1) and 33.7% in children (95% CI, 26.9–41.3). Nowadays, decreased renal perfusion, nephrotoxic medications [2] and sepsis [3] are considered as main causes for AKI. Among of them, contrast, which is widely used in enhanced radiation technology and interventional techniques, has attracted much attention from clinical investigators. Contrast-induced nephropathy (CIN) is characterized by rapid loss of kidney function after contrast administration, and it not only prolongs hospitalization but also associates with increased mortality. Although sensitivity C-reactive protein (SCr) has been used to define CIN, it is not an ideal indicator because of its limited effects in early diagnosis of CIN. Furthermore, SCr levels may be susceptible to some factors such as age, muscle mass, catabolism, and so on.

Neutrophil gelatinase-associated lipocalin (NGAL) is a 25-KD protein which expresses markedly in injured epithelial cells, and plays a significant role in attenuating apoptosis and promoting proliferation [4]. Devarajan firstly discovered that NGAL mRNA expression increased in animal kidney when AKI occurred [5]. Subsequently, a series of research on AKI also revealed that NGAL levels were high in both serum and urine [4,6,7]. Fibroblast growth factor 23 (FGF23) produced in bone was considered as regulator of phosphate metabolism on the kidneys, and was found to be increased with nephropathy progression in CKD population [8,9]. Existing studies also showed that circulating FGF23 rapidly increased after AKI in the context of rhabdomyolysis, use of folic acid, cardiac surgery and intensive care units [10–13]. Based on above research background, we suspect serum NGAL and FGF23 may be elevated in cases of CIN.

In order to explore clinical significance of serum NGAL and FGF23 for early diagnosis of CIN, the study focused on serum levels of these two markers at various time-points in patients undergoing PCI, and found out the optimal diagnostic model for CIN.

## Materials and methods

### Study population

The study was performed on 202 patients who underwent PCI due to coronary heart disease (CHD) from April 2015 to September 2015 in Xiangya Hospital of Central South University. The exclusion criteria were listed as follows: patients who use nephrotoxic drugs, diuretics or biguanides in perioperative period; patients with other contrast exposure within 2 weeks; patients with malignancy, acute and chronic infection uncontrolled, serious liver and pulmonary disease or severe kidney disfunction (estimated glomerular filtration rate (eGFR) <30 mL/min/1.73 m^2^); patients with instability hemodynamics. The investigation was approved by local institutional ethics committee (IRB approval number 201412444). Informed consent was received from all subjects before the procedure.

### Study design

Related data of all patients were obtained, such as baseline demographic and clinical characteristics, laboratory data and angiographic characteristics. Serum samples were collected for measuring levels of NGAL and FGF23 before and 0, 1, and 2 days after PCI. Serum NGAL was evaluated by automatic analyzer using immunoturbidimetry (PIEN TZE HUANG, China). Serum FGF23 was measured by intact FGF23 ELISA (R&D, America).

CIN is defined as the increment in SCr concentration >0.5 mg/dL or a minimum of 25% increase from baseline within 72 h after contrast administration in the light of European Society of Urogenital Radiology (ESUR) definition [14]. However, other causes induced AKI should be excluded. Based on the definition of CIN, all patients were classified into two groups, namely, CIN group and non-CIN group.

### Statistical analysis

Statistical analysis was carried out using the SPSS statistical package, version 20.0 (SPSS Inc, Chicago, IL). For the continuous variables, they were expressed as mean ± SD or median and interquartile range. If the variables were consistent with normal distribution, the differences of means were compared using unpaired *t* test. Otherwise, the comparison was analyzed by Mann-Whitney *U* test and Friedman *M* test. With respect to categorical variables, they are presented as counts and percentages, and the differences between two groups were compared by means of chi-square test. Receiver-operating characteristic (ROC) analysis was conducted to determine the cutoff value of serum NGAL and FGF23 in early diagnosis of CIN. And Spearman rank correlation test was used to correlate SCr, eGFR with NGAL, and FGF23, respectively. Logistic prediction models were established in StataSE 14 by a series of clinical parameters. Under the process of statistical analysis, Two-tailed values of *p* < .05 were regarded as statistically significant.

## Results

### Baseline clinical characteristics

In total 202 CHD patients with a mean age of 59.95 ± 10.56 were included, of which 165 patients (81.68%) were male. 30 (14.85%) patients developed CIN, but none of them received hemodialysis during hospitalization. The characteristics between CIN group and non-CIN group were compared which specific comparison results were listed in Tables 1–3. It could be seen that ratio of diabetes mellitus (DM), CKD and pre-admission administration of statins were significantly higher in patients who developed CIN after PCI. Patients had higher systolic blood pressure (SBP) and accepted greater volume of contrast in CIN group.

**Table 1.** Baseline demographic and clinical characteristics.

Variable	CIN		*p*
	No (*n* = 172)	Yes (*n* = 30)	
Age, years	59.58 ± 10.79	62.56 ± 8.54	.187
Male, *n* (%)	141 (82%)	24 (80%)	.796
Body mass index, kg/m^2^	23.85 (21.80,25.21)	24.06 (22.49,26.02)	.544
Current smokers, *n* (%)	82 (48%)	14 (47%)	.919
Hypertension, *n* (%)	107 (62%)	18 (60%)	.818
DM, *n* (%)	37 (22%)	12 (40%)	**.036**
CKD, *n* (%)	11 (6.4%)	6 (20.0%)	**.043**
Cardiac function levels III–IV, *n* (%)	33 (19%)	4 (13%)	.807
Prior PCI, *n* (%)	23 (13%)	2 (7%)	.466
Prior myocardial infarction, *n* (%)	24 (14%)	3 (10%)	.767
Systolic blood pressure, mmHg	129.42 ± 21.69	139.30 ± 22.29	**.023**
Diastolic blood pressure, mmHg	77.58 ± 12.44	80.17 ± 14.48	.306
Left ventricular diameter, mm	51.63 ± 5.94	50.32 ± 10.25	.416
Urine output within 24 h after PCI, ml PCIlml	1530 (1200,2130)	1480 (1240,2100)	.647
Prehospital medications, *n* (%)			
Aspirin	119 (69.1%)	15 (50.0%)	.065
ß-blocker	73 (42.4%)	7 (23.3%)	.076
Calcium channel blocker	40 (23.3%)	5 (16.7%)	.574
ACEI/ARB	61 (35.5%)	7 (23.3%)	.277
Statins	90 (34.9%)	6 (20.0%)	**.002**

Definitions: Current smokers were those who smoke daily at the last 6 months. Hypertension was defined as systolic blood pressure ≥140 mmHg and/or diastolic blood pressure ≥90 mmHg, at least two measurements, or the use of antihypertensive drugs. DM was defined as fasting blood glucose ≥7.0 mmol/L, or random blood glucose ≥11.1 mmol/L, at least two measurements, or the use of antidiabetes drugs. CKD was defined as eGFR <60 mL/min or abnormal urine elements more than 3 months. Bold number *p* < .05.

**Table 2.** Laboratory data.

Variable	CIN		*p*
	No (*n* = 172)	Yes (*n* = 30)	
White blood cell, *10^9^/L	7.76 ± 2.52	7.88 ± 2.21	.794
Platelet, *10^9^/L	207.57 ± 66.37	212.93 ± 62.19	.668
Hemoglobin, g/L	133.80 ± 16.40	131.20 ± 19.30	.470
Fasting plasma glucose, mmol/L	5.18 (4.74,6.34)	5.98 (4.90,7.22)	.078
SCr, umol/L	95.3 (85.0,108.0)	96.6 (87.6,112.0)	.124
eGFR, ml/min/1.73 m^2^	88.20 (75.69,100.93)	87.30 (74.00,101.28)	.197
Uric acid, umol/L	373.16 ± 97.53	367.10 ± 105.30	.217
Blood urea nitrogen, mmol/L	5.47 ± 1.66	5.45 ± 1.39	.934
Cystatin-C, mg/L	0.82 (0.71,1.02)	0.83 (0.76,1.04)	.199
Triglyceride, mmol/L	1.64 (1.21,2.42)	2.18 (1.15,3.11)	.189
Total cholesterol, mmol/L	4.29 (3.63,5.19)	5.05 (4.21,5.44)	.066
HDL, mmol/L	1.12 ± 0.28	1.10 ± 0.28	.643
LDL, mmol/L	2.80 ± 0.95	3.16 ± 0.97	.062
hsCRP, mg/L	2.78 (0.70,12.57)	3.13 (1.56,26.44)	.247
Fibrinogen, g/L	3.25 (2.71,3.96)	3.11 (2.73,3.81)	.659
Lactic dehydrogenase, U/L	210.0 (172.4,357.5)	220.0 (179.2,411.0)	.449
Creatine kinase, U/L	96.25 (67.95,222.80)	94.60 (68.25,232.40)	.873
Creatine kinase isoenzyme, U/L	19.90 (15.13,32.48)	18.60 (13.45,26.05)	.567
Myohemoglobin, ug/L	32.65 (24.43,44.80)	31.50 (22.05,69.65)	.806

Abbreviations: eGFR: estimated glomerular filtration rate, it was assessed by modified Modification of Diet in Renal Disease (MDRD) equations. HDL: high-density lipoprotein cholesterol. LDL: low-density lipoprotein cholesterol. hsCRP: high sensitivity C-reactive protein.

**Table 3.** Angiographic characteristics.

Variable	CIN		*p*
	No (*n* = 172)	Yes (*n* = 30)	
Type of contrast			.296
Iohexol	76 (42.9%)	11 (36.7%)	
Iodixanol	56 (32.6%)	8 (26.7%)	
Iopromide	40 (23.3%)	11 (36.7%)	
Contrast volume, mL	150 (120, 162.5)	160 (137.5,190)	**.041**
Number of stents used			.132
1, *n* (%)	56 (32.6%)	14 (46.7%)	
2, *n* (%)	64 (37.2%)	5 (16.7%)	
3, *n* (%)	45 (26.2%)	10 (33.3%)	
4, *n* (%)	7 (4.1%)	1 (3.3%)	
Gensini score	80 (59,107.5)	82.5 (63,114.25)	.533

Definition: Gensini score is a scoring system for determining the severity of CHD, which depends on the degree of luminal narrowing and the geographical importance of its location. Bold number *p* < .05.

### Changes in SCr, eGFR, serum NGAL, and FGF23

The levels of SCr, eGFR, serum NGAL, and serum FGF23 at various time points were listed in Table 4. Compared vertically, SCr and eGFR significantly increased after 2 days in two groups (*p* < .01). Although both serum NGAL and serum FGF23 reached peak 1 day after PCI. In CIN group, it appeared a remarkable rise in serum NGAL after 1 and 2 days and in serum FGF23 1 and 2 days after PCI (*p* < .01) comparing with the baseline values. Although the same trend was also displayed in non-CIN group (1 day after PCI, *p* < .01), the range was much smaller. Compared horizontally, the differences in SCr and eGFR between these two groups were distinct at 2 and 3 days after PCI (*p* < .01), and the differences in serum NGAL levels were significant after 1 and 2 days (*p* < .01). However, serum FGF23 in CIN group was prominently higher only 1 day after PCI in comparison to non-CIN group (*p* < .01).

**Table 4.** Serum levels of SCr, NGAL, and FGF23.

	Non-CIN group (*n* = 172)	CIN group (*n* = 30)
SCr (umol/L)		
Baseline	95.3 (85, 108)	96.6 (87.6, 112)
1 day after PCI	96.4 (86.3, 108.7)	97.2 (88.2, 116.4)
2 days after PCI	98.0 (87.5, 111.0)**	110.65 (101.2, 143.5)**##
3 days after PCI	100 (89.1, 111.2)**	113.3 (104.1, 155.8)**##
eGFR(ml/min/1.73m^2^)		
Baseline	88.20 (75.69, 100.93)	87.30 (74.00, 101.28)
1 day after PCI	87.49 (75.02, 99.94)	86.72 (73.85, 100.26)
2 days after PCI	85.56 (74.19, 97.32)**	75.34 (58.73, 85.21)**##
3 days after PCI	83.97 (72.82, 95.37)**	70.70 (50.71, 79.04)**##
Serum NGAL (ng/mL)		
Baseline	90 (77, 111.75)	88 (75, 108.5)
0 h after PCI	92.5 (77, 110)	89 (76.75, 111)
1 day after PCI	100.5 (86, 123)**	140.5 (119, 164.5)**##
2 days after PCI	93.5 (79, 111.75)	116.5 (91.5, 152)**##
Serum FGF23 (ng/L)		
Baseline	108.32 (94.46, 125.28)	111.75 (85.13, 138.68)
0 h after PCI	112.54 (95.56, 128.62)	113.4 (87.32, 143.84)
1 day after PCI	115.78 (99.29, 136.47)**	140.93 (108.32, 175.63)**##
2 days after PCI	112.96 (97.34, 128.95)	123.55 (94.58, 151.94)**

** *p* < .01 versus baseline. ##*p* < .01 compared among groups at the same time.

### Significance of serum NGAL and FGF23 in early diagnosis of CIN

**Figure 1.**
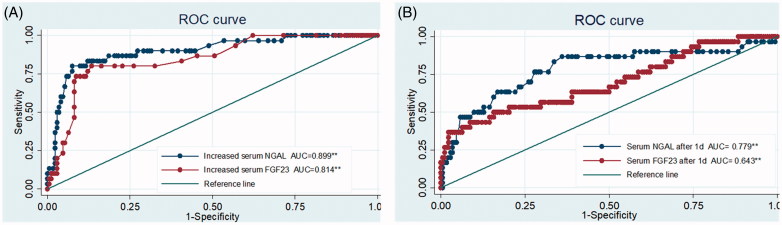
ROC curve of serum NGAL and FGF23, increment percentage between 1 day after PCI and baseline (A), absolute value 1 day after PCI (B). **p < .01.

To evaluate the diagnostic performance of serum NGAL and FGF23 for detecting CIN, the increment percentage of these two markers between 1 day after PCI and baseline was used to plot ROC curve (shown in Figure 1(A) and Table 5). The area under the curve (AUC) of relative values in serum NGAL was 0.899 (95% CI: 0.834–0.964, *p* = .000), and the optimum cutoff was 49% (sensitivity = 80.0%, specificity = 92.4%). The AUC in serum FGF23 was 0.814 (95% CI: 0.733–0.894, *p* = .000); the optimum cutoff was 20% (sensitivity = 73.3%, specificity = 87.6%). Similarly, Figure 1(B) revealed significance of absolute values 1 day after PCI for CIN diagnosis.

**Table 5.** Diagnostic characteristics of serum NGAL and FGF23 for CIN.

	AUC (95% CI)	Cut point	Sensitivity (%)	Specificity (%)
Serum NGAL				
Increment percentage	0.899 (0.834-0.964)	49%	80.0	92.4
Absolute value after 1 day	0.779 (0.675-0.882)	111.50 (ng/mL)	86.7	63.0
Serum FGF23				
Increment percentage	0.814 (0.733-0.894)	20%	73.3	87.6
Absolute value after 1 day	0.643 (0.520–0.766)	139.22 (ng/L)	50.0	80.6

### Correlation between SCr, eGFR, and serum NGAL, serum FGF23

**Figure 2.**
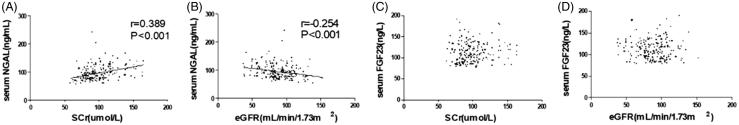
Correlation between SCr and serum NGAL (A), between eGFR and serum NGAL (B), between SCr and serum FGF23 (C), between eGFR and serum FGF23 (D).

The correlation between SCr, eGFR, and serum NGAL, serum FGF23 were shown in Figure 2. We can see that serum NGAL was positively correlated with SCr (*r* = 0.389, *p* < .001), and was negatively correlated with eGFR (*r* = –0.254, *p* < .001). Although the data in Figure 2(C,D) is relatively fragment which explains that there was no evident correlation between serum FGF23 and SCr, eGFR. The same procedure was also performed in 17 CKD patients no significant correlations were presented as well.

### Logistic prediction models for CIN

**Figure 3.**
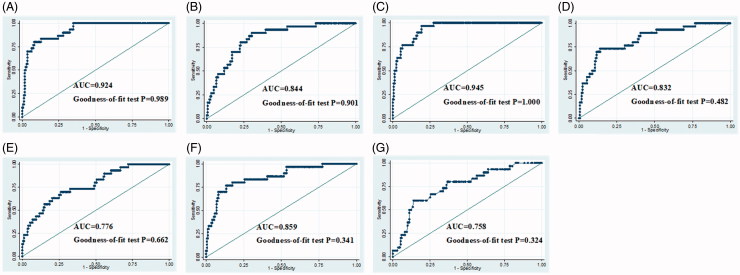
A-G: Shown are clinical models from models A–G.

Based on the foregoing univariate logistic regression analysis, the variable DM, CKD, SBP, and statins were greatly associated with CIN. To explore better ways of CIN recognition, various logistic prediction models were further built where took DM, CKD, SBP, and statins as four constant independent variable and serum NGAL and serum FGF23 were considered as optional variable. According to above principle, seven clinical models were established by StataSE 14. The components of each model were shown in Table 6 and the corresponding AUC curves were shown in Figure 3. Among seven models, Model G was taken as a compared model which just covered DM, CKD, SBP, and statins. As statistics go, the AUC of Model G was 0.758 and its goodness-of- fit *p* values was .324. Although the AUC of Models A, B, D, E, and F were 0.924, 0.844, 0.832, 0.776, and 0.859, respectively; the according goodness-of- fit *p* values were .989, .901, .482, .662, and .341. Obviously, the discrimination and calibration of models would increase no matter which optional variables taking into account. In these models, Model C consisted of increment percentage of serum NGAL, increment percentage of serum FGF23, DM, CKD, SBP, and statins, its AUC was 0.945 and its goodness-of- fit *p* values was 1.000, which had the highest discrimination and calibration.

**Table 6.** Clinical models.

	Model A	Model B	Model C	Model D	Model E	Model F	Model G
Constant	–12.778	–11.502	–19.947	–7.518	–6.614	–10.514	–4.120
X1	1.050	0.826	1.162	1.015	0.776	1.084	0.787
X2	0.974	1.071	0.932	0.443	0.819	0.317	0.953
X3	0.020	0.020	0.020	0.020	0.018	0.020	0.019
X4	–0.838	–1.270	–0.842	–1.119	–1.190	–1.130	–1.361
X5	6.115		5.566				
X6		6.123	6.674				
X7				0.026		0.027	
X8					0.024	0.027	

X1-X8: DM, CKD, SBP, statins, increment percentage of serum NGAL, increment percentage of serum FGF23, serum NGAL after 1 day, serum FGF23 after 1 day, respectively.

## Discussion

With the general application of coronary angiography and coronary interventional treatment, CIN, which is a potential complication, should not be neglected. Previous studies have reported that the risk of CIN in patients with normal renal function is low (1–2%) [15]. However, the incidence may be as high as 25% in patients with preexisting renal impairment or with combination of certain risk factors, such as CKD, DM, advanced age, and nephrotoxic drugs [16]. CIN after PCI portends a very high risk of death, both in hospital and in the long run [17]. Therefore, early diagnosis and treatment of CIN should be placed as number-one priority. SCr serves as the monitoring marker to detect CIN in current guidelines [18]. Nevertheless, SCr is unable to reflect kidney injury without delay. Thus, this is a major finding that both serum NGAL and FGF23 change prior to SCr in patients developed CIN.

According to recent studies, NGAL is seen as a promising new biomarker for AKI [4]. Serum NGAL rose as early as 2 h after contrast exposure, and it reached peak at 24 h after PCI [6]. Two meta-analyses, in 2009 [19] and 2015 [20], all revealed that serum NGAL worked well in early diagnosis of CIN. Considering their findings, the study payed more attention to serum levels of NGAL 1 day after PCI. Results demonstrated that the serum NGAL levels significantly increased after 1 day ahead of SCr. Furthermore, if increment percentage between 1 day after PCI and baseline was over 49%, serum NGAL had high sensitivity and specificity in CIN diagnosis, absolute values of serum NGAL after 1 day were good predictors for CIN as well. In contrast, relative values were superior to absolute values in detecting CIN at the time of 1 day after PCI. This superiority is more likely due to inapparent differences of serum NGAL between CIN group and non-CIN group at baseline and their minor changes after procedure. NGAL expressed boost in AKI plays a critical role in reducing renal tubular epithelial cell apoptosis by inhibiting the activation of caspase-3 [21,22]. The study also proved that serum NGAL inversely correlated with eGFR. So NGAL will be a new biomarker for early identifying CIN.

In terms of FGF23, it is well known that FGF23 acts as a hormone which regulates calcium and phosphorus metabolism [23]. What’s more, it also independently associates with left ventricular hypertrophy in individuals with CKD [24]. Recently, a number of researchers demonstrated that serum FGF23 rose clearly in patients who developed AKI. Survey conducted by Christov [12] showed that there was a rapid, early increase in serum FGF23 levels in animal models with toxin-induced AKI. FGF23 levels were also observed 15.9-fold increase at 24-h postoperatively in humans with AKI following cardiac surgery. In this study, it was the first time to detect FGF23 in CIN setting, serum FGF23 levels reached peak 1 day after PCI, but it was only slightly increase, which was different from previous reports. Two reasons may explain this phenomenon. One is that the setting in which AKI occurs has impact on serum FGF23 levels; the other is that the maximum value of FGF23 is observed in other time points within 1 day. Further researches should be conducted to explore this question. As shown in Table 5, relative values of serum FGF23 played some roles in CIN diagnosis, while absolute values did not result in well diagnostic performance. It needs more studies to evaluate whether serum FGF23 is applicable for early detection of CIN. A prospective study included 859 patients revealed that preoperative FGF23 level was independently predictor of postoperative AKI in the context of elective cardiac surgery [25], and other study in 19 children discovered that relative risk of developing AKI was 2.0 when preoperative FGF23 level was >86 RU/mL (*p* = .033) [26]. The logistic prediction models in our study also revealed that serum FGF23 detection after 1 day could improve the efficiency in early CIN diagnosis.

Efforts to change the clinical course of CIN have substantially failed because there are no effective therapies currently [27]. In this case, we have no choice but to prevent. So, it is necessary for clinical staff to evaluate the risk of CIN before contrast administration. Clinical models with serum NGAL and serum FGF23 showed high discrimination and calibration; therefore, our study suggested both serum NGAL and serum FGF23 could optimize the clinical models in early identifying CIN.

## Limitations

The study had only been conducted in one hospital, further study of larger population and multi-center need to be performed to validate the results. In addition, time points after PCI when serum was collected were too few to reflect the accurate changing trends of biomarkers. The study referred to patients with normal or moderately depressed kidney function, which may explain the phenomenon that serum levels of both NGAL and FGF23 were lower than other studies before. ESUR definition of CIN has lower threshold than those used in the risk, injury, failure, loss and end-stage kidney disease (RIFLE) criteria or the KDIGO definition. Thus it is susceptible to indicate CIN and bears the risk of overestimation in our study, which may have negative impact on evaluating the diagnostic efficient of serum NGAL and FGF23. Finally, CKD patients made up a very small proportion, thus it was not easy to explore the two markers in subjects with CKD or not.

## Conclusions

Consequently, serum levels of NGAL and FGF23 in CIN patients change significantly prior to the variation of SCr. They may have certain value in early diagnosis of CIN. Among these two markers, NGAL is better. Besides, clinical model including DM, CKD, SBP, increment percentage of serum NGAL, and serum FGF23 after 1 day may work well in early diagnosis of CIN.

## Ethical approval

All procedures performed in studies involving human participants were in accordance with the ethical standards of the institutional research committee at which the studies were conducted (IRB approval number 201412444) and with the 1964 Helsinki declaration and its later amendments.

## Informed consent

Informed consent was obtained from all individual participants included in the study.
